# Untargeted Metabolomics Based on Ultra-High-Performance Liquid Chromatography Coupled with Quadrupole Orbitrap High-Resolution Mass Spectrometry for Differential Metabolite Analysis of Pinelliae Rhizoma and Its Adulterants

**DOI:** 10.3390/molecules29092155

**Published:** 2024-05-06

**Authors:** Jing Wang, Jie Cui, Ziyi Liu, Yang Yang, Zhan Li, Huiling Liu

**Affiliations:** Institute of Medicinal Plant Development, Chinese Academy of Medical Sciences & Peking Union Medical College, Beijing 100193, China; jwang@implad.ac.cn (J.W.); jcui@implad.ac.cn (J.C.); zyliu@implad.ac.cn (Z.L.); yangyang@implad.ac.cn (Y.Y.); zli@implad.ac.cn (Z.L.)

**Keywords:** Pinelliae Rhizoma, adulterants, UHPLC-Q-Orbitrap HRMS, untargeted metabolomics

## Abstract

The present study investigates the chemical composition variances among Pinelliae Rhizoma, a widely used Chinese herbal medicine, and its common adulterants including *Typhonium flagelliforme*, *Arisaema erubescens*, and *Pinellia pedatisecta*. Utilizing the non-targeted metabolomics technique of employing UHPLC-Q-Orbitrap HRMS, this research aims to comprehensively delineate the metabolic profiles of Pinelliae Rhizoma and its adulterants. Multivariate statistical methods including PCA and OPLS-DA are employed for the identification of differential metabolites. Volcano plot analysis is utilized to discern upregulated and downregulated compounds. KEGG pathway analysis is conducted to elucidate the differences in metabolic pathways associated with these compounds, and significant pathway enrichment analysis is performed. A total of 769 compounds are identified through metabolomics analysis, with alkaloids being predominant, followed by lipids and lipid molecules. Significant differential metabolites were screened out based on VIP > 1 and *p*-value < 0.05 criteria, followed by KEGG enrichment analysis of these differential metabolites. Differential metabolites between Pinelliae Rhizoma and *Typhonium flagelliforme*, as well as between Pinelliae Rhizoma and *Pinellia pedatisecta*, are significantly enriched in the biosynthesis of amino acids and protein digestion and absorption pathways. Differential metabolites between Pinelliae Rhizoma and *Arisaema erubescens* are mainly enriched in tyrosine metabolism and phenylalanine metabolism pathways. These findings aim to provide valuable data support and theoretical references for further research on the pharmacological substances, resource development and utilization, and quality control of Pinelliae Rhizoma.

## 1. Introduction

Pinelliae Rhizoma, the dried tuber of *Pinellia ternata* (Thunb.) Breit., belonging to the *Araceae* family, possesses a pungent taste and warm property, exhibiting toxicity along with therapeutic effects such as antiemetic, phlegm-resolving, and antitumor activities [1,2,3]. Modern pharmacological studies have revealed its diverse pharmacological actions, including antibacterial, anti-inflammatory, antiepileptic, and anticancer properties [4,5,6]. There are six species within the genus *Pinellia*, predominantly distributed in Eastern Asia, with five species found in China, primarily in the southern and northern regions. As a traditional Chinese medicinal herb with a history of over two thousand years, Pinelliae Rhizoma has been utilized not only in traditional Chinese medicine clinical formulations but also as a constituent in various Chinese patent medicines [7,8].

However, recent years have witnessed a continuous increase in demand, coupled with a decline in wild resources and severe lagging in cultivation techniques, leading to a shortage of genuine Pinelliae Rhizoma in the market, and consequently the proliferation of adulterants [9,10]. Common adulterants include *Pinellia pedatisecta* Schott [11], which is of the same genus but a different species with high yield and a low price, as well as *Typhonium flagelliforme* (Lodd.) Blume [12] and *Arisaema erubescens* (Wall.) Schott [13,14], which belong to different genera within the same family. The mixing of adulterants severely compromises the clinical efficacy of Pinelliae Rhizoma. Therefore, effective discrimination between Pinelliae Rhizoma and common adulterants is crucial for ensuring its quality, clinical efficacy, and resource conservation.

Pinelliae Rhizoma, derived from the dried underground tuber, poses challenges in distinguishing genuine from adulterated products solely based on visual inspection after removal of the leaves and other organs, leading to medication confusion and exacerbating safety and quality issues. Liquid chromatography or its multipurpose mass spectrometry techniques are commonly reported methods for chemical composition analysis of traditional Chinese medicine (TCM) [15,16,17,18]. However, chromatographic methods rely on reference standards and struggle to detect low-abundance chemical constituents in TCM, resulting in limited quantitative analysis and inadequate reflection of the metabolome [19]. Metabolomics, a discipline studying endogenous metabolites and their variations within organisms, constitutes an integral part of systems biology [20,21,22]. Its focus includes small molecule metabolites with relative molecular weights below 1000, encompassing organic acids, amino acids, lipids, and other compounds [23]. TCM authentication is pivotal for quality control [24,25], with metabolomics serving as a tool for quality assessment based on metabolic information similarity. While metabolomics techniques have been widely applied in comparative studies of TCM quality [26,27,28,29], scarce literature exists on metabolomics investigations of Pinelliae Rhizoma and its common adulterants.

Therefore, this study aimed to comprehensively determine the chemical compositions and metabolic profiles of *Pinellia ternata* (Thunb.) Breit. [Banxia (BX)] and its common adulterants including *Typhonium flagelliforme* (Lodd.) Blume [Shuibanxia (SBX)], *Arisaema erubescens* (Wall.) Schott [Tiannanxing (TNX)], and *Pinellia pedatisecta* Schott [Huzhangnanxing (HZNX)]. This research is performed via untargeted metabolomics analysis based on ultra-high-performance liquid chromatography coupled with quadrupole Orbitrap high-resolution mass spectrometry (UHPLC-Q-Orbitrap HRMS). Principal component analysis (PCA) and orthogonal partial least squares discriminant analysis (OPLS-DA) were employed for metabolite classification, exploring methods for identification based on differences in metabolite categories. Volcano plot analysis was utilized to ascertain upregulated and downregulated differential compounds, while Kyoto Encyclopedia of Genes and Genomes (KEGG) pathway analysis revealed disparities in metabolic pathways of the compounds, allowing for the identification of significantly enriched pathways. The outcomes of this investigation are poised to establish an effective means of discerning Pinelliae Rhizoma from its adulterants, thereby furnishing both empirical evidence and theoretical underpinnings for the examination of the pharmacological constituents inherent to Pinelliae Rhizoma, resource development and utilization endeavors, and quality control measures.

## 2. Results and Analysis

### 2.1. Overall Analysis of Metabolite Composition in Pinelliae Rhizoma and Its Adulterants

**Figure 1:**
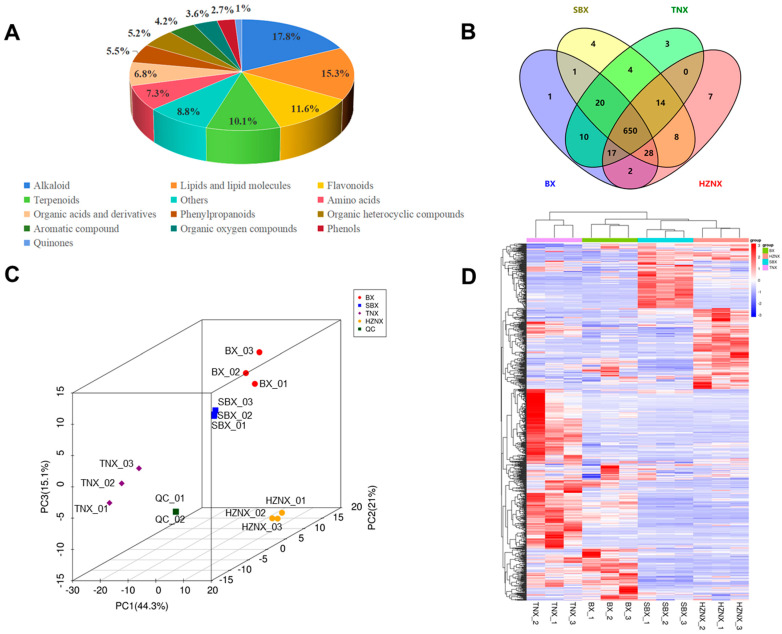
(**A**) The distribution of metabolic substances in BX, SBX, TNX, and HZNZ. (**B**) Venn diagram of metabolite distribution in BX, SBX, TNX, and HZNZ. (**C**) 3D PCA of metabolites identified from BX, SBX, TNX, and HZNZ. (**D**) HCA exhibiting correlation among BX, SBX, TNX, and HZNZ.

A total of 769 metabolites belonging to 13 categories were identified in Pinelliae Rhizoma and its three adulterants. Among these, alkaloids accounted for 17.8%, lipids and lipid molecules for 15.3%, flavonoids for 11.6%, terpenoids for 10.1%, amino acids for 7.3%, organic acids and derivatives for 6.8%, phenylpropanoids for 5.5%, organic heterocyclic compounds for 5.2%, aromatic compounds for 4.2%, organic oxygen compounds for 3.6%, phenols for 2.7%, quinones for 1%, and other compound classes for 8.8% (Figure 1A, Supplementary Table S1). BX was found to contain 729 metabolites, while SBX, TNX, and HZNX contained 729, 718, and 726 metabolites, respectively. A total of 650 metabolites were common to Pinelliae Rhizoma and its three adulterants. Only one unique metabolite was identified in BX, which is physalin L. The four unique metabolites identified in SBX are chinenoside III, goyasaponin III, astragaloside IV, and so on. The three unique metabolites identified in TNX are isorhamnetin-3-O-neohesperidine, typhaneoside, and quercetin 3-(2Gal-apiosylrobinobioside). Moreover, the seven unique metabolites identified in BX includes licoricone, macranthoidin B, goyaglycoside h, and so on (Figure 1B).

PCA is an unsupervised pattern recognition method that effectively extracts the most significant information from complex data by reducing its dimensionality. Utilizing PCA for pattern recognition of metabolite differences between Pinelliae Rhizoma and its adulterants resulted in PCA score plots (Figure 1C). Each biological replicate of the samples clustered together, showing clear separation from the quality control (QC) samples, indicating minimal variation among the intra-group samples, thus ensuring the repeatability and reliability of the laboratory procedures. Between genuine and adulterant samples, PC1 contributed 44.3%, PC2 contributed 21%, and PC3 contributed 15.1%, revealing distinct patterns among genuine and three adulterant samples with significant separation, indicating significant differences in their metabolic phenotypes.

Hierarchical cluster analysis (HCA) was performed on all samples of Pinelliae Rhizoma and its adulterants (Figure 1D). Pinelliae Rhizoma and its three adulterants clustered separately into distinct groups, each subdivided into four regions, indicating significant differences in chemical composition among Pinelliae Rhizoma and its adulterants. The established untargeted metabolomics approach effectively characterizes the chemical features of Pinelliae Rhizoma and its adulterants.

### 2.2. OPLS-DA and Permutation Test Analysis

**Figure 2:**
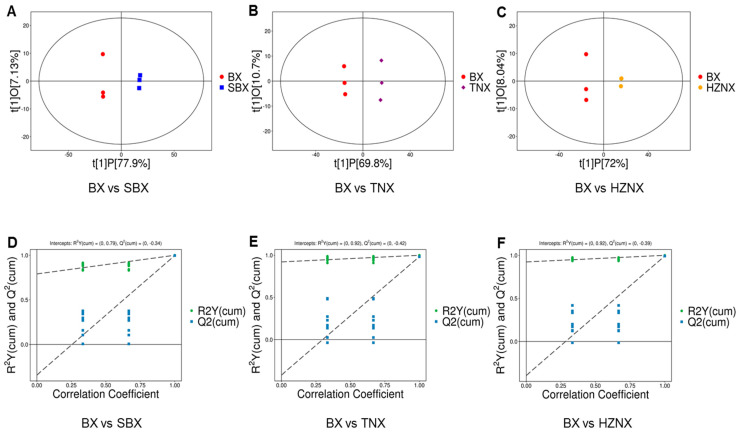
(**A**–**C**) OPLS-DA score plot of BX vs. SBX, BX vs. TNX, and BX vs. HZNX, respectively. (**D**–**F**) OPLS-DA permutation plot of BX vs. SBX, BX vs. TNX, and BX vs. HZNX, respectively.

OPLS-DA is a supervised pattern recognition method in multivariate statistical analysis. It conducts orthogonal partial least squares discriminant analysis on all low relative molecular weight metabolites in the samples to filter differential variables by eliminating unrelated differences. Comparisons were made pairwise among BX, SBX, TNX, and HZNX. The OPLS-DA score plots (Figure 2A–C) revealed that in the comparisons of BX vs. SBX, BX vs. TNX, and BX vs. HZNX, PC1 contributed 77.9%, 69.8%, and 72%, respectively, while PC2 contributed 7.13%, 10.7%, and 8.04%, respectively. Each comparison group exhibited distinct separation trends, indicating significant differences in metabolites among the three groups of samples.

The validation of the OPLS-DA models (Table 1) indicated that for BX vs. SBX, R2X = 0.85, R2Y = 1, and Q2 = 0.997; for BX vs. TNX, R2X = 0.804, R2Y = 0.999, and Q2 = 0.982; and for BX vs. HZNX, R2X = 0.8, R2Y = 1, and Q2 = 0.992. All evaluation parameters of the OPLS-DA models exceeded 0.5, and Q2 > 0.9, indicating well-constructed models with reliable predictive capabilities.

To verify the reliability of the OPLS-DA models, permutation tests were conducted. Samples were randomly permuted multiple times to shuffle the grouping, and corresponding OPLS-DA models were established to calculate their R2Y and Q2 values. The results were plotted as scatter plots, with two dashed lines representing the regression lines of R2Y and Q2 (see Figure 2D–F). The results indicated that the slopes of the R2Y regression lines for the comparisons of BX vs. SBX, BX vs. TNX, and BX vs. HZNX were all positive, and the R2Y values were generally located above the Q^2^ values. This suggests that the models were meaningful and not overfitting. Differential metabolites could be selected for analysis based on their VIP values.

### 2.3. Selection and Analysis of Differential Metabolites between Pinelliae Rhizoma and Its Adulterants

**Figure 3:**
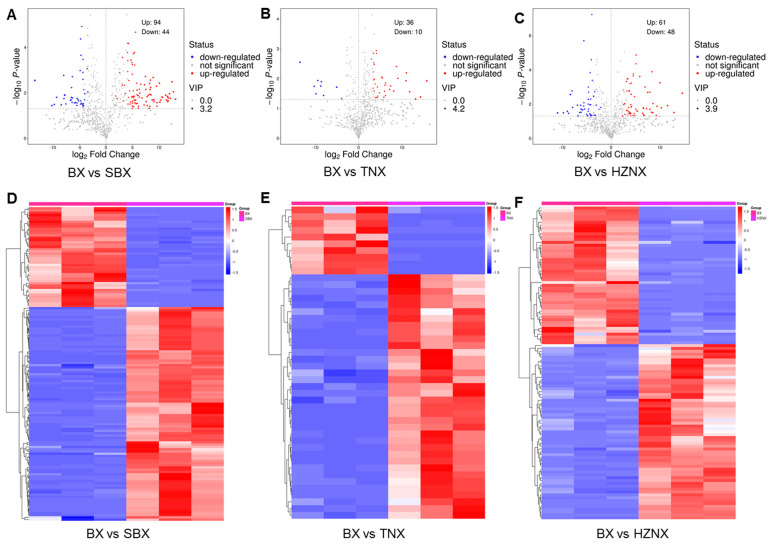
(**A**–**C**) Volcano plot of differential metabolites among BX vs. SBX, BX vs. TNX, and BX vs. HZNX, respectively. (**D**–**F**) Heatmap clustering exhibiting correlation among BX vs. SBX, BX vs. TNX, and BX vs. HZNX, respectively.

Based on the results of the OPLS-DA, metabolites with VIP > 1 and *p*-value < 0.05 were selected as significantly differential metabolites. To visually reflect the overall trend of metabolite content differences between the two groups and the statistical significance of metabolite differences, volcano plots of differential metabolites were generated (Figure 3A–C). In the comparison of BX vs. SBX, 138 differential metabolites were identified, with 94 upregulated (68.1%) and 44 downregulated (31.9%) metabolites. In BX vs. TNX, 46 differential metabolites were identified, with 36 upregulated (78.3%) and 10 downregulated (21.7%) metabolites. In BX vs. HZNX, 109 differential metabolites were identified, with 61 upregulated (56%) and 48 downregulated (44%) metabolites. The table of differential metabolites is provided in Supplementary Table S2, showing the relative abundance trends of each group, with red indicating upregulated metabolites and blue indicating downregulated metabolites.

For a convenient and intuitive observation of metabolite change patterns, heatmap clustering was performed on significant differential metabolites, as shown in Figure 3D–F and Supplementary Table S3. The red area represents high-expression regions of differential metabolites, while the blue area represents low-expression regions. In the comparison of BX vs. SBX, differential metabolites of BX were mainly concentrated in the red high-expression area, while those of SBX were mainly concentrated in the blue low-expression area. BX exhibited significantly lower expression levels of 44 differential metabolites compared to SBX, while significantly higher expression levels were observed for 94 differential metabolites, mainly comprising terpenoids, flavonoids, and lipids and lipid molecules. Similarly, in BX vs. TNX and BX vs. HZNX comparisons, BX showed higher expression levels of differential metabolites mainly comprising flavonoids and alkaloids, while TNX and HZNX exhibited lower expression levels. Overall, the comparison revealed a closer similarity between BX and TNX in terms of differential metabolites. Furthermore, differential metabolite heatmap analysis demonstrated differences in the content and types of differential metabolites among Pinelliae Rhizoma and its three adulterants, suggesting the significance of differential metabolites in distinguishing Pinelliae Rhizoma from its adulterants.

### 2.4. Venn Diagram Analysis of Differential Metabolites

**Figure 4:**
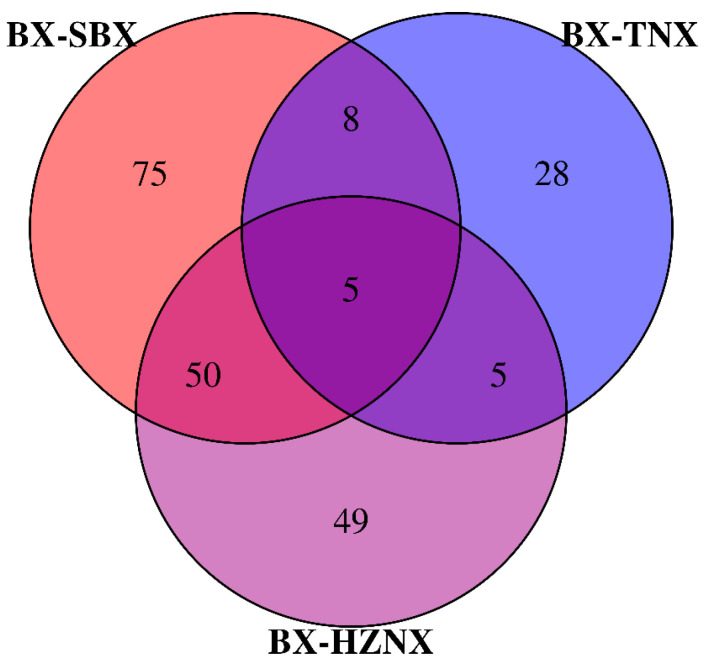
Venn plot of the number of differential metabolites among BX vs. SBX, BX vs. TNX, and BX vs. HZNX.

The results of the Venn diagram analysis of differential metabolites are shown in Figure 4 and Supplementary Table S4. A total of 220 differential metabolites were identified among Pinelliae Rhizoma and its adulterants, with 75, 28, and 49 specific differential metabolites in the BX vs. SBX, BX vs. TNX, and BX vs. HZNX groups, respectively. There were five common differential metabolites among the three groups, including dioscoretine, jervine, terfenadine, and so on. A total of 13 differential metabolites were shared between BX vs. SBX and BX vs. TNX, while 55 were shared between BX vs. SBX and BX vs. HZNX, and 10 were shared between BX vs. TNX and BX vs. HZNX. These results indicate that the metabolites of BX and TNX are relatively similar. However, while both are used in traditional medicine, they have different efficacies and should not be used interchangeably.

### 2.5. K-Means Clustering Analysis of Differential Metabolites in Pinelliae Rhizoma and Its Adulterants

**Figure 5:**
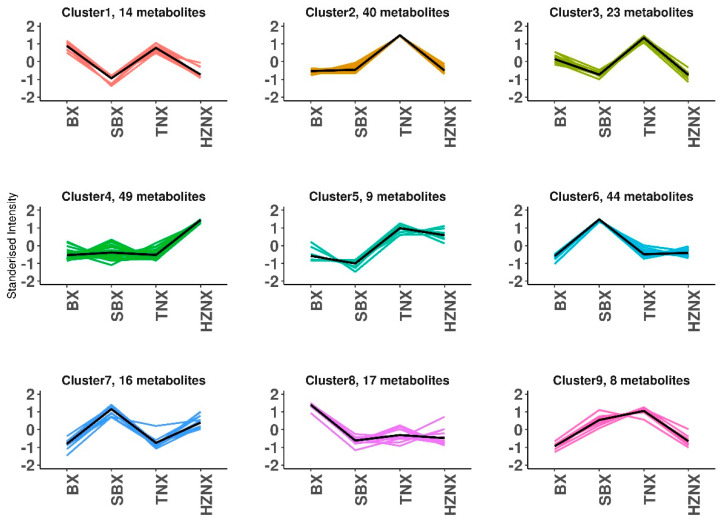
K-means clustering of differential metabolites profiles.

In order to investigate the relative abundance changes of metabolites in Pinelliae Rhizoma and its adulterants in different groups, the relative abundance of the 220 identified differential metabolites obtained according to the screening criteria was standardized using *z*-score, followed by K-means clustering analysis. As shown in Figure 5, the 220 differential metabolites in Pinelliae Rhizoma and its three adulterants were divided into 9 subclasses. Subclasses 2, 3, 5, and 9 contained 40, 23, 9, and 8 metabolites, respectively, with TNX having higher standardized values than the other three. Subclasses 6 and 7 contained 44 and 16 metabolites, respectively, with SBX having higher standardized values than the other three. Subclasses 1 and 8 contained 14 and 17 metabolites, respectively, with BX having higher standardized values than the other three. Subclass 4 contained 49 metabolites, with HZNX having higher standardized values than the other three. Detailed K-means data are provided in Supplementary Table S5.

### 2.6. KEGG Pathway Enrichment Analysis of Differential Metabolites in Pinelliae Rhizoma and Its Adulterants

**Figure 6:**
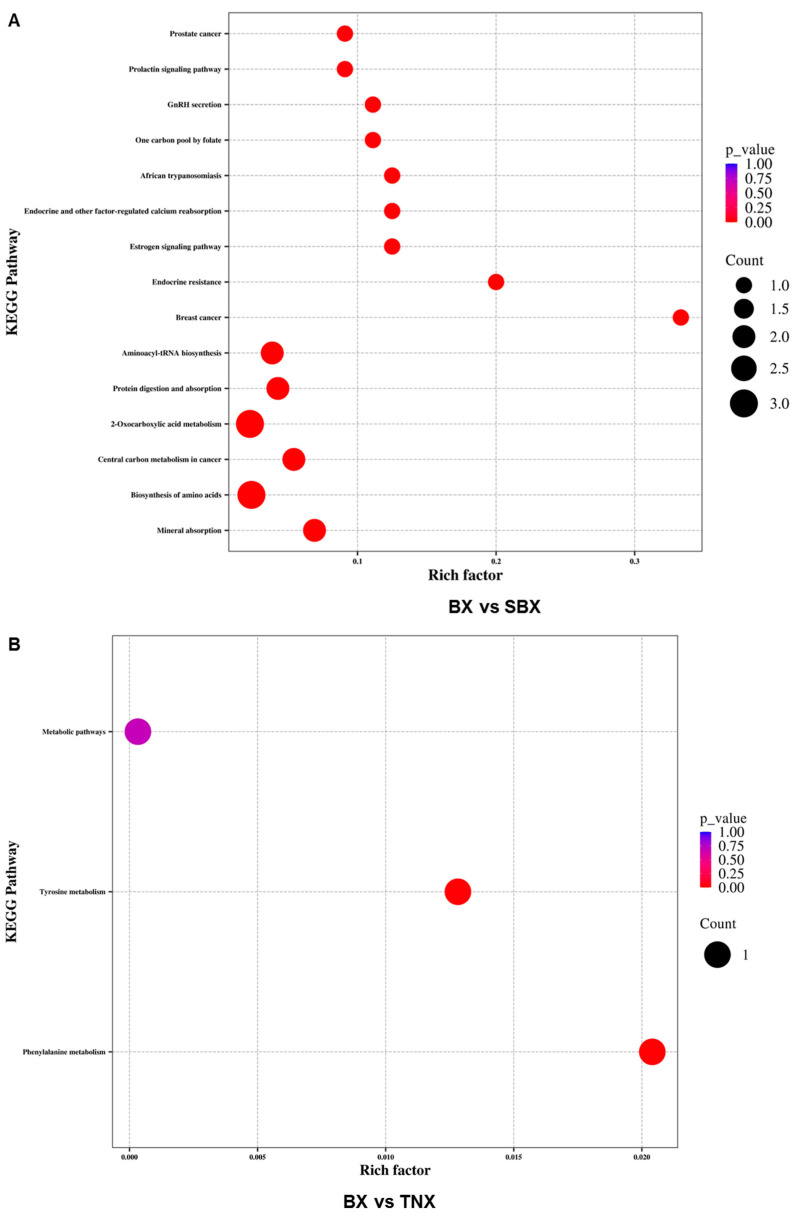

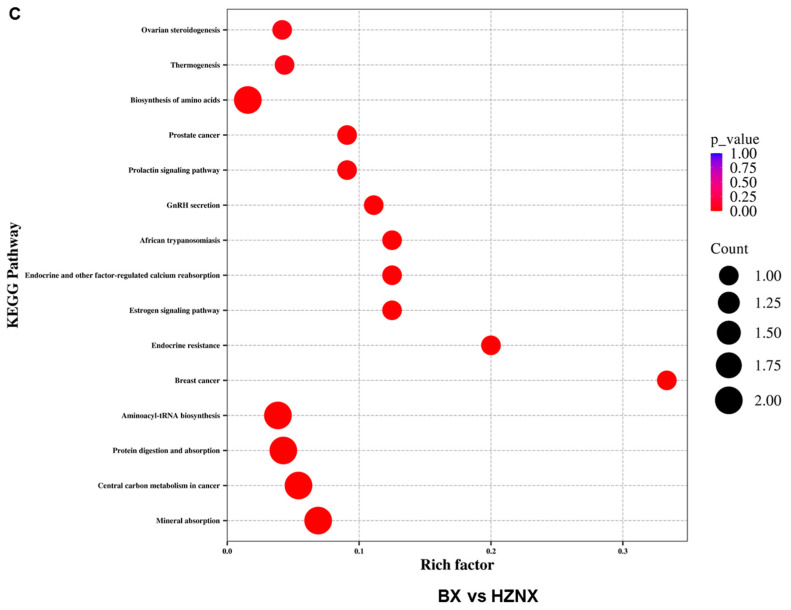
(**A**–**C**) KEGG enrichment map of differential metabolites among BX vs. SBX, BX vs. TNX, and BX vs. HZNX, respectively.

The growth process of plants is regulated by multiple substances and reactions, making it a highly complex metabolic process. It cannot be evaluated solely based on the abundance of certain substances. Therefore, further analysis of metabolic pathways is needed. We used the KEGG database to annotate the differential accumulated metabolites in each comparison group and conducted enrichment analysis on the annotated results to obtain the pathways of differential accumulated metabolites (Figure 6, Supplementary Table S6).

For the comparison between BX and SBX, differential metabolites were distributed across 41 metabolic pathways. Different metabolites were predominantly enriched in the biosynthesis of amino acids, 2-Oxocarboxylic acid metabolism, aminoacyl-tRNA biosynthesis, protein digestion and absorption, mineral absorption, and central carbon metabolism in cancer. For the comparison between BX and HZNX, differential metabolites were distributed across 34 metabolic pathways. Different metabolites were mainly enriched in the biosynthesis of amino acids, aminoacyl-tRNA biosynthesis, protein digestion and absorption, central carbon metabolism in cancer, and mineral absorption. There were overlapping differential enrichment pathways between BX vs. SBX and BX vs. HZNX, such as the biosynthesis of the amino acids pathway and the protein digestion and absorption pathway. The former pathway included 18 differential metabolites, i.e., aspartate, arginine, L-glutamine, L-serine, ornithine, L-tryptophan, tyrosine, 2-ketobutyric acid, leucine, L-histidine, L-proline, L-asparagine, citric acid, valine, threonine, 2-oxoadipic acid, L-isoleucine, and o-acetylserine. The latter pathway contained 16 differential metabolites, i.e., aspartate, arginine, L-glutamine, L-serine, L-tryptophan, tyrosine, leucine, L-histidine, L-proline, L-asparagine, valine, threonine, histamine, L-isoleucine, indole, and tyramine.

For the comparison between BX and TNX, differential metabolites were distributed across three metabolic pathways. Different metabolites were mainly enriched in tyrosine metabolism and phenylalanine metabolism. The tyrosine metabolism pathway included eight differential metabolites, which are succinate, tyrosine, fumaric acid, tyramine, gentisic acid, 4-hydroxyphenylacetic acid, maleic acid, and homovanillic acid. Meanwhile, the phenylalanine metabolism pathway included seven differential metabolites, which are succinate, tyrosine, fumaric acid, 4-hydroxyphenylacetic acid, 2-hydroxycinnamic acid, L-3-phenyllactic acid, and 2-phenylethanol.

## 3. Discussion

Pinelliae Rhizoma, a traditional Chinese medicinal herb, mainly contains alkaloids, organic acids, amino acids, flavonoids, and other chemical components, possessing significant medicinal value. Li et al. [30] conducted a comprehensive analysis employing high-performance liquid chromatography (HPLC) to simultaneously assess eight components aimed at evaluating the quality of Pinelliae Rhizoma and its various processed products. Their investigation revealed the presence of twelve active constituents within Pinelliae Rhizoma. Notably, their findings suggested that *β*-sitosterol could potentially exert a more pronounced influence on quality evaluation compared to other active ingredients. In addition, Zhang et al. [10] employed species-specific nucleotide assays to meticulously assess 56 Pinelliae Rhizoma products available in the Chinese market, encompassing medicinal slices, powders, and Chinese patent medicines. Their comprehensive analysis unveiled a concerning trend, with approximately 66% of the examined products exhibiting adulteration. The predominant adulterants identified included HZNX (present in 57% of the analyzed samples), followed by TNX (9%), *Typhonium giganteum* (2%), and SBX (2%).

Metabolomics technology can comprehensively analyze the overall chemical composition of traditional Chinese medicines and has been widely applied in the quality evaluation of Chinese medicinal materials [26,27,28,29]. In this study, based on UHPLC-Q-Orbitrap HRMS untargeted metabolomics technology, we investigated the metabolites of BX and its common adulterants, SBX, TNX, and HZBX. A total of 769 metabolites were identified, with alkaloids accounting for 17.8%, lipids and lipid molecules 15.3%, flavonoids 11.6%, terpenoids 10.1%, amino acids 7.3%, and organic acids and their derivatives 6.8%. Based on the OPLS-DA results, significant differential metabolites were screened using the criteria of VIP > 1 and *p*-value < 0.05. For BX vs. SBX, 138 differential metabolites were screened, with 94 upregulated metabolites (68.1%) and 44 downregulated metabolites (31.9%). For BX vs. TNX, 46 differential metabolites were screened, with 36 upregulated metabolites (78.3%) and 10 downregulated metabolites (21.7%). For BX vs. HZNX, 109 differential metabolites were screened, with 61 upregulated metabolites (56%) and 48 downregulated metabolites (44%).

Through KEGG enrichment analysis of differential metabolites, the differential metabolites in BX vs. SBX and BX vs. HZNX were significantly enriched in the biosynthesis of amino acids and protein digestion and absorption pathways, mainly including amino acid compounds such as aspartate, arginine, L-glutamine, and L-proline, which are essential or non-essential amino acids, with L-proline being a component of Pinelliae Rhizoma’s irritant. Differential metabolites in BX vs. TNX were mainly enriched in tyrosine metabolism and phenylalanine metabolism pathways, mainly including organic acids and their derivatives such as succinate, fumaric acid, gentisic acid, and maleic acid. Sun et al. [31] found that organic acids in Pinelliae Rhizoma have antitussive and expectorant effects, constituting the main pharmacologically-active substances in Pinelliae Rhizoma.

Secondary metabolites of plants are closely related to key enzymes and transcriptional regulatory factors in synthetic metabolic pathways. By analyzing metabolic pathways, the reasons for phenotypic differences in the research objects can be identified. Pinelliae Rhizoma and its adulterants have differences in chemical composition, and it is speculated that the genes significantly enriched in the above pathways may be the reasons for the different phenotypes of Pinelliae Rhizoma and its adulterants.

## 4. Materials and Methods

### 4.1. Instruments and Reagents

The analytical instrumentation comprised the Orbitrap Exploris 120 quadrupole-Orbitrap high-resolution mass spectrometer (Thermo Fisher Scientific, Waltham, MA, USA) coupled with the Vanquish UHPLC ultra-high-performance liquid chromatography system (Thermo Fisher Scientific, USA). Chromatographic separation was achieved using an Acquity UPLC BEH C18 column (2.1 mm × 100 mm, 1.7 μm particle size, Waters, Milford, MA, USA). Solvents employed included methanol, acetonitrile, and formic acid (LC-MS grade, CNW Technologies, Dusseldorf, Germany), alongside purified water.

### 4.2. Sample Collection

Samples of Pinelliae Rhizoma and its three common adulterants were collected in August 2022 from the Banxia cultivation base in Xihe Town, Gansu Province, China. Each sample was collected with three replicates. The samples were identified by Dr. Linchun Shi, a researcher at the Institute of Medicinal Plant Development, Chinese Academy of Medical Sciences. They are the dried tubers of BX, SBX, TNX, and HZNX.

### 4.3. Sample Preparation

The test medicinal herbs underwent a meticulous processing regimen, beginning with freeze-drying, followed by pulverization into powder samples using conditions set at 60 Hz for 120 s. Subsequently, 100 mg of each powdered sample was meticulously weighed and combined with 500 μL of extraction solution composed of methanol and water in a ratio of 4:1 (*v*/*v*). The resultant mixture underwent vortexing for 30 s, homogenization at 45 Hz for 4 min, and subsequent sonication for 1 h in an ice-water bath. Following this, the sample was allowed to stand at −40 °C for 1 h before being subjected to centrifugation at 12,000 rpm for 15 min. The supernatant was then meticulously filtered through a 0.22 μm microporous membrane to yield the test solution.

### 4.4. Chromatographic Conditions

Chromatographic separation was executed utilizing an Acquity UPLC BEH C18 column (2.1 mm × 100 mm, 1.7 μm particle size). The mobile phase consisted of 0.1% formic acid in water (A) and 0.1% formic acid in acetonitrile (B). A gradient elution program was meticulously devised as follows: 0–5 min, 5–15% B; 3.5–6 min, 15–30% B; 6–6.5 min, 30% B; 6.5–12 min, 70% B; 12–12.5 min, 70% B; 12.5–25 min, 100% B; 25–30 min, 5% B. The flow rate was maintained at 0.4 mL/min, with the column temperature set at 30 °C, and an injection volume of 5 μL was employed.

### 4.5. Mass Spectrometry Conditions

Mass spectrometric analysis was executed utilizing a heated electrospray ionization (HESI) source in both positive and negative ion modes. Specifically, the spray voltages were meticulously adjusted to 5.5 kV for positive mode and 4.0 kV for negative mode. The sheath gas flow rate was rigorously maintained at 9 L/min, while the auxiliary gas flow rate was set at 3 L/min. Operating parameters such as the ion transfer tube and vaporization temperatures were set at 350 °C. A Full MS/dd-MS^2^ scan mode was employed for secondary mass spectrometry testing, maintaining a resolution of 60,000 for Full MS scans and 15,000 for dd-MS^2^ scans. The scan range spanned from *m*/*z* 100 to 1500. Collision energies under the NCE mode were precisely set at 16, 38, and 42 eV.

### 4.6. Data Analysis Methods

Data Processing and Multivariate Pattern Analysis: The raw data underwent conversion into the mzXML standard format employing ProteoWizard. Subsequent processes, including peak identification, extraction, comparison, and conformity assessment, were carried out utilizing the R by XCMS package. Metabolites were annotated by referencing an in-house MS^2^ database (BiotreeDB). Principal Component Analysis (PCA) and Orthogonal Partial Least Squares Discriminant Analysis (OPLS-DA) were performed using SIMCA 16.0.2 software. Evaluation of the OPLS-DA model included a 7-fold cross-validation to compute R2 (model fitness) and Q2 (predictive ability), followed by a permutation test (200 iterations). Metabolites exhibiting variable importance in the projection (VIP) scores > 1 and *p*-values < 0.05 (determined by Student’s *t*-test) were deemed potential differential metabolites in this investigation.

Metabolic Pathway Analysis: The metabolic enrichment pathways associated with putative differential metabolites influencing cellular metabolism were investigated using MetaboAnalyst (http://metpa.metabolomics.ca accessed on 5 January 2024 for all the websites) and the Kyoto Encyclopedia of Genes and Genomes (KEGG) pathway database (http://www.genome.jp/kegg/ accessed on 5 January 2024). Through a comprehensive analysis of pathways linked to the identified differential metabolites, the most statistically significant pathways associated with metabolic variances were determined.

## 5. Conclusions

To briefly summarize, in this study, UHPLC-Q-Orbitrap HRMS untargeted metabolomics technology was employed in conjunction with multivariate statistical techniques to devise a robust approach for discriminating between Pinelliae Rhizoma and its adulterants. The findings offer substantial empirical evidence and theoretical insights, thereby facilitating the exploration of the pharmacological substance foundation, resource exploration and exploitation, as well as quality assurance of Pinelliae Rhizoma.

## Figures and Tables

**Table 1:** Parameters of the OPLS-DA model.

Mode	R2X (cum)	R2Y (cum)	Q2 (cum)
BX vs. SBX	0.85	1	0.997
BX vs. TNX	0.804	0.999	0.982
BX vs. HZNX	0.8	1	0.992

## Data Availability

Data are available in the supplementary materials.

## Supplementary Materials

The following supporting information can be downloaded at: https://www.mdpi.com/article/10.3390/molecules29092155/s1, Table S1: Overall metabolites; Table S2: Differential metabolites; Table S3: Significant differential metabolites for heatmap clustering analysis. Table S4: Differential metabolites for the Venn diagram analysis; Table S5: K-means data. Table S6: Differential accumulated metabolites for the KEGG analysis.
